# Feasibility of AI-driven disease surveillance systems at international airports in sub-Saharan Africa: a narrative review

**DOI:** 10.3389/fpubh.2026.1834330

**Published:** 2026-07-07

**Authors:** Elizabeth Ezekiel Malingumu, Dotto Daniel Kisendi, Rasabs Wende Pierre Kabore, Onacis Yeremon Guerde, Moses Mugerwa, Qun He

**Affiliations:** 1 Department of Preventive Services, Ministry of Health, Dodoma, Tanzania; 2 National Health Insurance Fund, Directorate of Membership Services, Ministry of Health, Morogoro, Tanzania; 3 Dori Health District, Ministry of Health, Dori, Sahel Region, Burkina Faso; 4 Outpatient Care Center of the Bangui Community Hospital, Ministry of Health, Bangui, Central African Republic; 5 School of Public Health, Southern Medical University, Guangzhou, China; 6 Institute for Global Health, Southern Medical University, Guangzhou, China; 7 Dermatology Hospital, Southern Medical University, Guangzhou, China; 8 School of International Education, Southern Medical University, Guangzhou, China

**Keywords:** airports, artificial intelligence, disease surveillance, early warning systems, public health, sub-Saharan Africa

## Abstract

**Background:**

Artificial Intelligence (AI) has the potential to enhance disease surveillance, particularly at international airports, by improving the early detection and response to infectious diseases. This narrative review assesses the feasibility of implementing AI-driven disease surveillance systems at international airports in SSA.

**Methods:**

A comprehensive search of academic databases was conducted to identify relevant studies and policies. The review synthesized findings and categorized them into three thematic areas: AI effectiveness, ethical and privacy concerns, and infrastructure and capacity gaps.

**Results:**

The review suggests that implementing AI-driven disease surveillance systems at SSA international airports may be feasible in principle, although the available evidence is largely conceptual and policy-oriented rather than derived from empirical deployment at airports. Realizing this potential would likely depend on first addressing critical barriers such as inadequate data quality, insufficient infrastructure, and a shortage of trained personnel. These challenges might be mitigated through targeted investments in digital infrastructure, workforce capacity-building, and the establishment of clear regulatory frameworks to support ethical AI deployment.

**Conclusion:**

This study suggests that AI-driven disease surveillance could meaningfully strengthen public health security at international airports in Sub-Saharan Africa, provided that critical challenges in infrastructure, privacy, and regulation are addressed. The review offers a preliminary, evidence-informed framework rather than confirmation of operational feasibility. Country-specific feasibility studies and pilot implementations will be essential to test these systems under real-world conditions and to inform a possible shift toward more resilient, data-driven health infrastructures.

## Introduction

Artificial intelligence (AI) is the technology that enables computers and machines to simulate human intelligence, allowing them to learn, understand, and solve problems (1). AI in healthcare refers to the use of intelligent machines, through algorithms or sets of rules, designed to mimic human cognitive functions such as learning and problem-solving, to enhance medical practice and healthcare delivery (2, 3). The application of AI in disease surveillance at international airports has become increasingly significant as it enhances early detection and response to emerging infectious diseases (4). By automating data analysis, AI systems enhance the efficiency and precision of disease surveillance (5).

The enhanced mobility of individuals and goods across the globe through international travel has accelerated the global spread of infectious diseases (6). Air transport, in particular, has been at the core of the transmission of diseases such as COVID-19, SARS, and Ebola (7). The World Health Organization (WHO) has reported that the ease and frequency of international travel have amplified the spread of diseases such as influenza, tuberculosis, and COVID-19 (6, 8), necessitating the need to enhance global disease surveillance (8, 9).

Despite the critical role airports play in controlling the spread of diseases, in most Sub-Saharan African (SSA) countries, disease surveillance systems are often underdeveloped and under-resourced (10). The majority of airports lack advanced surveillance technologies, with surveillance efforts primarily reliant on manual health checks and traditional methods such as temperature screening (11). However, these methods are often inadequate for detecting diseases that do not exhibit immediate symptoms, such as certain infectious diseases during their incubation period. Moreover, the infrastructure for real-time monitoring is typically insufficient, with airports often facing challenges such as limited technological capacity, inadequate data integration, and a shortage of trained personnel (12).

Given these limitations, integrating AI-driven disease surveillance systems could provide a transformative solution by enhancing the accuracy and speed of disease detection at airports (13). Unlike traditional methods like temperature screening, which are ineffective during the incubation period of diseases and prone to human error, AI technologies can analyze multimodal data (e.g., thermal imaging, travel history, audio cough analysis) in real-time (14). This enables the detection of subtle, pre-symptomatic patterns indicative of illness, greatly enhancing accuracy. Furthermore, AI systems eliminate the delay inherent in manual data entry and analysis by providing continuous, automated monitoring (15). They can process large datasets from passenger screening processes instantly, detect anomalies, and offer predictive insights to identify outbreaks much earlier than traditional systems allow (16). However, the transition to AI-based systems at SSA airports faces numerous challenges, such as the need for robust digital infrastructure, capacity-building programs for personnel, and the development of regulatory frameworks that support AI deployment in public health settings (17).

Airport-based surveillance differs in several important respects from community- or facility-based public health surveillance, and these differences shape both the opportunities and the constraints for AI deployment. First, airports function as controlled points of entry at which a large, mobile, international population is screened at the precise moment of cross-border transmission risk, rather than within a defined resident catchment population. Second, screening at airports is acutely time-sensitive: decisions must be reached within seconds per passenger to avoid disrupting passenger flow, whereas most community and clinical surveillance operate on longer timescales. Third, point-of-entry surveillance is governed jointly by public health authorities and by civil aviation and border agencies under the International Health Regulations (2005), creating a multi-agency operating environment with distinct legal, security, and data-governance requirements. These features generate airport-specific challenges around throughput, passenger consent, interoperability with existing entry-screening workflows, and aviation security that are not fully captured by the broader literature on AI in public health surveillance. Accordingly, the present review treats this broader literature as an indirect evidence base from which feasibility at SSA airports is inferred, while explicitly identifying the airport-specific evidence gap.

The purpose of this review is to evaluate the feasibility and potential impact of implementing AI-driven disease surveillance systems at airports in SSA. This review synthesizes existing studies on AI applications in airport disease surveillance to assess their feasibility in SSA. The review draws on global case studies to identify key challenges and opportunities, which inform the subsequent discussion on how these findings can be applied to SSA. It examines the current infrastructure, surveillance capacities, and operational challenges at SSA airports, highlighting barriers. Based on evidence from global studies, the review provides strategic recommendations to address these challenges.

## Methods

The review employed well-established databases such as PubMed, ScienceDirect, and Web of Science and prominent organizational websites such as WHO, Tanzania Civil Aviation Authority (TCAA), Tanzania Airport Authority (TAA), Tanzania Communications Regulatory Authority (TCRA), and Ministry of Health (MoH), Tanzania, to identify relevant studies and policies/guidelines. Articles were retrieved using a combination of keywords and Boolean operators. The searching phrases were (“Artificial Intelligence” AND “disease surveillance” OR “early warning systems”) OR (“Artificial Intelligence” AND “Public Health”). For replication, the study period was limited to articles published between 2019 and 2025, capturing the most recent advancements in the field. This approach ensures that the results are based on contemporary research and technological developments. In addition to academic articles, 9 policy documents were reviewed during the screening process. The 9 policy documents were identified and screened using predefined inclusion criteria. After screening, 7 policies were included in the study.

Articles were excluded if they were non-peer-reviewed, published before 2019, focused on non-health-related AI applications, or lacked direct relevance to public health surveillance. Furthermore, studies in languages other than English were also excluded.

**Figure 1:**
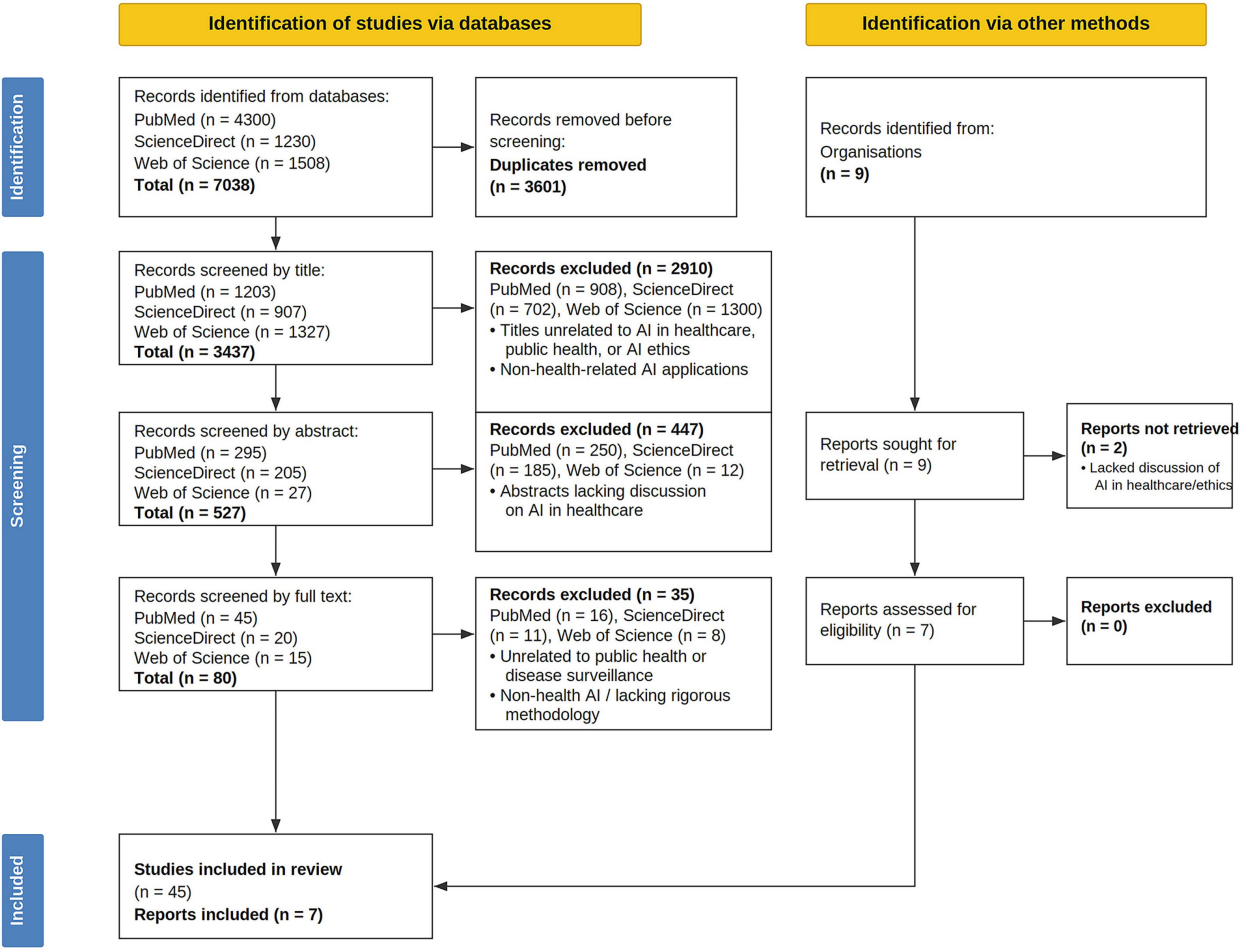
PRISMA flow diagram highlighting the selection process of the final studies included in this narrative review.

The screening process involved an initial removal of duplicates, followed by the identification of articles and policy documents pertinent to AI in disease surveillance at airports. Relevant studies were selected for full-text evaluation, while policy documents related to AI in disease surveillance were assessed for their relevance to the review. Articles that did not align with the research focus or lacked methodological rigor were excluded. This narrative review was informed by a structured search process, ensuring that the selected studies addressed key aspects of AI applications in public health, particularly in disease surveillance. The study selection process is summarized in Figure 1.

A narrative synthesis approach was employed to systematically summarize the findings of the included studies, which were categorized into three thematic areas, as shown in Table 1. These thematic areas were derived through a logical process of categorizing the findings based on the major themes that emerged from the data. Each study was reviewed for its focus, and the themes were constructed to reflect the key dimensions necessary for evaluating the feasibility of implementing AI systems at SSA international airports. These three thematic areas were; We note at the outset that few peer-reviewed studies evaluate AI-driven surveillance specifically at airports, and fewer still at SSA airports. This review therefore draws on the broader evidence base on AI in infectious-disease and public-health surveillance and reasons toward feasibility in the SSA airport context, while explicitly identifying the airport-specific evidence gap as a priority for future research.

**Table 1:** Thematic analysis of the included studies.

Thematic area	Citation	Title	Study characteristics	Objective	Key findings/conclusions	Limitations
AI effectiveness	Diallo et al. (2022) (19)	Artificial Intelligence Approaches to Predict COVID-19 Infection in Senegal	AI algorithms applied on a dataset of 12,727 individuals.	Develop predictive models for COVID-19 infection using AI algorithms.	Achieved 73% accuracy and AUROC of 0.69 in predictions.	Limited to a single geographic area, affecting generalizability.
	Hutchinson et al. (2023) (54)	Could it be Monkeypox? Use of AI-based Early Warning System to Monitor Rash and Fever Illness	Analyzed data through EPIWATCH AI system for syndrome detection.	Identify signals of rash and fever illness as potential Mpox outbreaks.	Significant increase in rash-like illnesses detected in 2022.	Relied on media reports, which may introduce bias.
	Zhao et al. (2024) (25)	AI for Science: Predicting Infectious Diseases	Reviewed literature on AI in public health and predictive modeling.	Explore the role of AI in predicting infectious diseases.	AI enhances accuracy and timeliness of predictions.	Did not address variability in data quality across regions.
	Ankolekar et al. (2024) (21)	Using AI and Predictive Modelling to Enable Learning Healthcare Systems for Pandemic Preparedness	Narrative review synthesizing insights from literature.	Assess the role of AI in enhancing pandemic preparedness.	Importance of integrating AI for improved pandemic management.	Lacked empirical data to support proposed models.
	Olaboye et al. (2024) (24)	Innovations in Real-Time Infectious Disease Surveillance Using AI and Mobile Data	Literature and case studies on AI and mobile data in surveillance.	Explore applications of AI and mobile data in disease surveillance.	AI can improve outbreak detection and response.	Limited focus on implementation challenges in different contexts.
	Tshimula et al. (2024) (27)	AI for Public Health Surveillance in Africa: Applications and Opportunities	Literature review on AI applications across Africa.	Investigate AI applications in public health surveillance in Africa.	AI enhances disease monitoring and health outcomes.	Limited infrastructure and skilled workforce in many regions.
	Sundermann et al. (2022) (26)	Whole-Genome Sequencing Surveillance and Machine Learning of the Electronic Health Record	Observational study combining WGS and ML for outbreak detection.	Evaluate effectiveness of EDS-HAT in outbreak detection.	Detected multiple outbreaks not identified by traditional methods.	Limited to specific pathogens and settings.
	McKee et al. (2025) (55)	The Power of Artificial Intelligence for Managing Pandemics: A Primer for Public Health Professionals	Narrative review of AI applications during COVID-19.	Explore AI applications in public health for pandemic management.	AI improves outbreak detection and resource allocation.	Ethical concerns and data privacy issues remain.
	Anjaria et al. (2023) (56)	Artificial Intelligence in Public Health: Revolutionizing Epidemiological Surveillance for Pandemic Preparedness and Equitable Vaccine Access	Narrative review of AI applications in public health.	Examine AI’s role in enhancing epidemiological surveillance and vaccine access.	AI improves outbreak prediction and healthcare delivery.	Ethical concerns regarding data privacy and algorithm bias.
	Simsek and Kantarci (2020) (57)	Artificial Intelligence-Empowered Mobilization of Assessments in COVID-19-Like Pandemics: A Case Study for Early Flattening of the Curve	Simulation-based approach using self-organizing feature maps (SOFM) to optimize mobile assessment deployment.	To propose an AI-driven strategy for mobilizing assessment agents during pandemics.	SOFM can significantly reduce unassessed populations in the early days of an outbreak.	Simulation results may not reflect real-world complexities; limited to one case study area.
	Marley et al. (2024) (58)	Collective Intelligence-Based Participatory COVID-19 Surveillance in Accra, Ghana: Pilot Mixed Methods Study	Mixed methods study; participants traded on COVID-19 predictions using the Prediki platform.	To assess the feasibility and acceptability of an IM surveillance system to monitor COVID-19 outcomes in Accra, Ghana.	IM surveillance was feasible and acceptable; active trading encouraged by peer influence.	Low participation rate (20%); reliance on self-reported data.
	Weeks et al. (2023) (59)	Using Artificial Intelligence to Advance Public Health	Commentary based on literature review; discusses AI applications in public health.	To explore AI’s potential in enhancing public health, particularly in LMICs.	AI can improve healthcare access, diagnostic accuracy, and support public health initiatives.	Ethical concerns on AI transparency and accountability; potential job displacement.
	Giansanti (2022) (60)	Artificial Intelligence in Public Health: Current Trends and Future Possibilities	Editorial reviewing AI’s applications and trends in public health; supported by literature search.	To summarize current trends and future possibilities of AI in public health.	AI significantly enhances diagnostics, personalized care, and healthcare efficiency.	Limited discussion on practical challenges in AI implementation in public health.
	He et al. (2023) (61)	Research on the Application of Artificial Intelligence in Public Health Management: Leveraging Artificial Intelligence to Improve COVID-19 CT Image Diagnosis	Proposed method for refining lesion labels using deep learning; validated on COVID-19 CT datasets.	To improve COVID-19 diagnosis efficiency through AI-based CT image analysis.	DLShelper significantly improves accuracy in lesion segmentation and severity grading.	Limited dataset size; manual labeling may introduce bias.
	Fiscal et al. (2022) (62)	COVID-19 Classification Using Thermal Images	Analyzed thermal video data with machine learning methods; features extracted from different body regions.	To investigate the role of thermal imaging for identifying SARS-CoV-2-infected individuals.	Thermal imaging showed limited sensitivity and specificity for COVID-19 detection.	Not sensitive enough for mass screening; further validation needed in diverse settings.
	Saponara et al. (2021) (63)	Implementing a real-time, AI-based, people detection and social distancing measuring system for COVID-19	Utilized YOLOv2 for deep learning, implemented on a low-cost embedded system (Jetson Nano).	To develop an AI system for detecting individuals and measuring social distancing using thermal images.	Achieved accuracy of 98.5% in detecting individuals and assessing social distancing.	Limited to specific environments; dependent on the quality of thermal images.
	Morgenstern et al. (2021) (64)	AI’s gonna have an impact on everything in society, so it has to have an impact on public health	Conducted in-depth semi-structured interviews with 15 experts in public health and AI; thematic analysis of transcripts.	To explore the potential impacts of AI on public health practice.	Identified opportunities and barriers for AI in public health; emphasized the need for high-quality data.	Small sample size may limit generalizability; reliance on self-reported data from experts.
	Ullah et al. (2022) (65)	Explainable artificial intelligence approach in combating real-time surveillance of COVID-19 pandemic from CT scan and X-ray images using ensemble model	Proposed a deep learning model using CNN for feature extraction, followed by an ensemble model for classification.	To establish an explainable AI system for COVID-19 detection using CT scans and X-ray images.	Achieved 98.5% accuracy with ensemble learning; demonstrated robustness in detecting COVID-19.	Dependence on quality of training data; computational intensity may limit application in low-resource settings.
	Bavli and Galea (2024) (66)	Key Considerations in the Adoption of Artificial Intelligence in Public Health	Opinion piece discussing the implications of AI in public health and potential biases.	To investigate the values and biases in AI technology affecting public health.	Emphasizes the need for transparency and ethical considerations in AI deployment.	Lacks empirical data to support claims; primarily theoretical discussion.
	Suvvari and Kandi (2024) (16)	Artificial intelligence enhanced infectious disease surveillance—A call for global collaboration	Letter to the editor summarizing the potential of AI in enhancing disease surveillance.	To advocate for global collaboration in AI for infectious disease surveillance.	AI can transform early detection and response to infectious diseases through better data analysis.	Lacks specific case studies or examples of successful implementation.
	Pezanowski et al. (2024) (67)	Artificial intelligence enhanced infectious disease surveillance—A call for global collaboration	Analyzed disease outbreaks using geospatial AI and machine learning; focused on malaria, cholera, and yellow fever.	To advocate for the use of AI in enhancing infectious disease surveillance in Africa.	Geographic proximity influences disease outbreaks; machine learning models predicted disease presence.	Limited to four diseases; data imbalances affected model accuracy.
	Aiello et al. (2020) (68)	Social media- and internet-based disease surveillance for public health	Review of digital surveillance methods, emphasizing algorithms and data sources like social media.	To investigate the potential of social media and internet data for enhancing public health surveillance.	Digital surveillance can capture unreported cases and trends; highlights the need for integration with traditional systems.	Ethical concerns regarding privacy and data accuracy; biases in digital data sources.
	Surianarayanan and Chelliah (2021) (69)	Leveraging Artificial Intelligence (AI) Capabilities for COVID-19 Containment	Review of literature on AI applications in COVID-19 management.	To explore the role of AI in addressing challenges posed by COVID-19.	AI can improve outbreak prediction, diagnostics, and resource allocation.	Limited empirical examples; reliance on literature without extensive case studies.
	Tanui et al. (2024) (70)	Artificial intelligence to transform public health in Africa	Qualitative analysis highlighting AI applications in surveillance, outbreak detection, and healthcare accessibility.	To discuss the integration of AI in public health surveillance and response in Africa.	AI can enhance disease detection and response in Africa, addressing healthcare workforce shortages.	Challenges include inadequate infrastructure and limited access to technology.
	Colubri et al. (2019) (71)	Machine-learning Prognostic Models From the 2014? 2016 Ebola Outbreak	Analysis of clinical data from Ebola treatment units, employing logistic regression and machine learning.	To develop and validate prognostic models for Ebola virus disease (EVD) using machine learning.	Developed robust models for predicting EVD outcomes; highlights the importance of data harmonization.	Data limitations due to incomplete records; reliance on specific datasets may limit applicability.
	Farooq et al. (2022) (23)	Predictors of disease outbreaks at continental scale in the African region	Utilized machine learning (XGBoost) and XAI (SHAP) to analyze eco-climatic data and predict disease outbreaks.	To identify eco-climatic drivers of West Nile virus outbreaks using AI and geospatial data.	Model showed strong predictive ability; identified key factors linked to WNV outbreaks.	Limited to WNV; may not account for other emerging infectious diseases.
	Sajid et al. (2022) (72)	SARS-CoV-2: Has Artificial Intelligence Stood the Test of Time?	Review of AI tools used for diagnosis, surveillance, and management of COVID-19.	To evaluate the effectiveness of AI applications during the COVID-19 pandemic.	AI tools aided in diagnosis and policy formulation; highlighted applications in contact tracing.	Concerns about privacy, ethical use, and algorithm reliability.
	McClymont et al. (2024) (73)	Internet-based Surveillance Systems and Infectious Diseases Prediction	Systematic review of studies on digital surveillance for infectious diseases from 2013–2024.	To evaluate trends in digital surveillance for infectious diseases over the last decade.	Internet-based surveillance enhances early detection of outbreaks; improves predictive modeling accuracy.	Limitations in data quality and access; potential for misinformation.
	Sukums et al. (2023) (48)	The Use of Artificial Intelligence-Based Innovations in the Health Sector in Tanzania: A Scoping Review	A scoping review of AI applications in Tanzania’s health sector, examining 18 studies related to AI’s role in disease prediction, diagnosis, and resource optimization.	To investigate the status, challenges, and opportunities for AI in the Tanzanian health system.	AI is effective in disease prediction (cholera) and diagnosis (malaria), optimizing healthcare resources. Machine learning techniques have been applied to enhance disease detection and response.	Poor data quality, lack of AI policies, and insufficient technical expertise in applying AI for disease surveillance.
Ethical and Privacy	Smidt and Jokonya (2021) (32)	The Challenge of Privacy and Security When Using Technology to Track COVID-19	Systematic Literature Review of 40 articles focusing on privacy concerns.	Explore implications of using technology for tracking COVID-19 and privacy concerns.	Highlighted tension between public health needs and privacy rights.	Focused primarily on technology without public engagement strategies.
	Dankwa-Mullan (2024) (74)	Health Equity and Ethical Considerations in Using Artificial Intelligence in Public Health	Commentary based on literature review discussing health equity and ethics.	Explore health equity and ethical issues in AI deployment.	Importance of equitable access to AI technologies in healthcare.	Lacked specific case studies illustrating principles discussed.
	Bharel et al. (2024) (75)	Transforming Public Health Practice with Generative Artificial Intelligence	Commentary discussing AI applications in public health functions.	Explore how generative AI can support public health functions.	Generative AI improves communication and decision-making insights.	Challenges in maintaining public trust and ensuring equity.
	Tang et al. (2020) (36)	Artificial Intelligence Plays an Important Role in Containing Public Health Emergencies	Review of AI applications in epidemic control.	Discuss the role of AI in COVID-19 management.	AI enhances detection, diagnosis, and management of diseases.	Limited discussion on long-term implications of AI use.
	Mudey et al. (2024) (37)	Artificial Intelligence in Healthcare With An Emphasis on Public Health	Commentary on AI applications in public health and primary care.	Explore AI advancements in public health.	Optimizes diagnostic processes and enhances delivery.	Ethical considerations and data privacy concerns remain.
	Webster and Neal (2024) (35)	Ethical Principles of Artificial Intelligence in Public Health	Correspondence discussing AI ethics and oversight.	Address ethical considerations of AI in public health.	Requires ethical oversight and transparency in AI applications.	Lack of cohesive ethical frameworks across countries.
	Murdoch (2021) (33)	Privacy and artificial intelligence: challenges for protecting health information in a new era	Discussed privacy issues, access, and control of patient data through qualitative analysis of existing literature.	To examine privacy concerns associated with AI in healthcare and propose regulatory measures.	Highlighted the need for robust data protection measures and maintaining patient agency.	Regulation may lag behind technology; reliance on private entities poses risks.
	Lekadir et al. (2025) (76)	FUTURE-AI: International Consensus Guideline for Trustworthy and Deployable Artificial Intelligence in Healthcare	This paper provides an international framework for ensuring AI in healthcare is deployed ethically, safely, and transparently. It emphasizes fairness, accountability, and explainability as key principles for trustworthy AI.	To offer a set of guidelines that ensure AI tools in healthcare are ethically sound, fair, and legally compliant, with a focus on transparency and privacy.	AI must be designed to avoid algorithmic bias, ensure data privacy, and maintain transparency in decision-making processes. Ethical AI deployment is essential to build trust among users, healthcare providers, and patients.	Lacks empirical validation of how these guidelines function in real-world settings. Still needs more specific case studies to address practical challenges.
Infrastructure and capacity	Jayatilleke (2020) (41)	Challenges in Implementing Surveillance Tools of High-Income Countries in Low- and Middle-Income Countries	Qualitative review of surveillance systems in LMICs.	Discuss barriers to implementing surveillance systems in LMICs compared to HICs.	Key barriers include resource limitations and lack of trained personnel.	Lacked specific examples of successful implementations in LMICs.
	Greco et al. (2024) (77)	AI-Enhanced Tools and Strategies for Airborne Disease Prevention in Cultural Heritage Sites	Review of AI technologies in epidemiological surveillance.	Explore AI applications in cultural heritage sites for disease prevention.	AI enhances air quality monitoring and pathogen detection.	Implementation challenges due to site-specific conditions.
	Otaigbe (2022) (43)	Scaling Up Artificial Intelligence to Curb Infectious Diseases in Africa	Opinion piece outlining AI applications in Africa.	Discuss potential of AI in combating infectious diseases.	AI can enhance diagnosis and healthcare accessibility.	Limited implementation in resource-poor settings.
	Mustafa et al. (2023) (42)	Digital Technologies to Enhance Infectious Disease Surveillance in Tanzania: A Scoping Review	Scoping review of literature on technologies for disease surveillance.	Summarize literature on mobile and computer-based technologies for disease surveillance.	Identified 13 technologies; many lacked interoperability.	Limited investment in community-based surveillance; fragmentation in implementation.
	Struelens et al. (2024)(78)	Real-Time Genomic Surveillance for Enhanced Control of Infectious Diseases and Antimicrobial Resistance	Review and recommendations for genomic surveillance in managing infectious diseases.	Advocate for genomic surveillance to manage infectious diseases and AMR.	Genomic surveillance is crucial for tracking pathogens.	Challenges in data sharing and integration across sectors.
	Andigema et al. (2024) (79)	Transforming African Healthcare with AI: Paving the Way for Improved Health Outcomes	Explore AI’s integration in African healthcare systems.	Review of AI applications and case studies in healthcare.	AI improves access and efficiency in healthcare delivery.	Limited infrastructure and training in AI application.
	Sun (2021) (80)	Adopting Artificial Intelligence in Public Healthcare: The Effect of Social Power and Learning Algorithms	Qualitative multi-case study of AI adoption in three hospitals in China.	Explore factors affecting AI adoption in healthcare from a social power perspective.	Quarantine is the most effective strategy; AI influenced by social power structures.	Limited hospital data, a staff-centric focus, and exclusion of policy factors constrain AI adoption studies. Its single-country (China) focus and lack of long-term analysis further limit generalizability.
	Fawole et al. (2023) (81)	COVID-19 surveillance in Democratic Republic of Congo, Nigeria, Senegal and Uganda: strengths, weaknesses and key lessons	Analyzed COVID-19 surveillance strategies in four African countries.	To analyze COVID-19 surveillance strategies and document lessons learned.	Identified strengths in leveraging existing systems but highlighted gaps in staffing and integration.	Limited generalizability due to context-specific findings.

A narrative synthesis was selected deliberately, in preference to a systematic review or meta-analysis, because of the nature of the research question and the available evidence. The question addressed here, whether AI-driven surveillance is feasible at SSA airports, and what would be required to achieve it is exploratory and spans several distinct domains: the technical performance of AI models, ethical and data-privacy considerations, health-system infrastructure, workforce capacity, and aviation and regulatory policy. A single answerable review question of the kind a systematic review is designed to resolve does not capture this breadth, whereas narrative synthesis is well suited to integrating conceptually heterogeneous evidence across disciplines. A meta-analysis was not possible: the relevant studies differ fundamentally in design (including narrative reviews, commentaries, simulation studies, diagnostic-accuracy studies, and policy documents) and report no common outcome measure, comparable populations, or shared endpoints that could be quantitatively pooled. For these reasons, a narrative approach offered the most appropriate means of mapping the current evidence, identifying cross-cutting barriers and enablers, and deriving an implementation and policy framework for an under-examined setting.

AI effectiveness in disease surveillance: These studies were grouped based on their focus on AI algorithms’ accuracy, predictive power, and generalizability in detecting infectious diseases.

Ethical and privacy concerns: These studies were grouped based on their examination of the ethical implications of AI-driven health surveillance, particularly regarding data privacy, security, and algorithmic bias.

Infrastructure and capacity gaps: These were grouped based on their analysis of the infrastructure and capacity challenges that hinder the successful implementation of AI systems in low and middle-income countries (LMICs) and the need for capacity-building programs to train healthcare professionals and airport staff in AI applications.

A formal quality appraisal was not conducted. Instead, studies were carefully selected based on their methodological rigor and relevance to the review’s objectives.

## Results

The findings from this review suggest that AI-driven disease surveillance systems have the potential to enhance disease detection accuracy and response times in airports, as suggested by early experience in resource-limited countries such as Senegal and the Seychelles (18, 19). However, the effectiveness of these systems is often constrained by challenges related to local data quality and infrastructure, which could limit their application in other SSA regions (20).

### AI effectiveness in disease surveillance

#### Accuracy and timeliness of predictions

Studies have shown that AI can boost the accuracy and speed of outbreak predictions, greatly improving disease surveillance and pandemic readiness (21). During the 2014–2016 Ebola outbreak, machine learning models built from clinical data proved effective in forecasting the outcomes of Ebola virus disease (EVD) (22). Furthermore, machine learning models effectively identified key eco-climatic drivers of West Nile virus outbreaks, demonstrating strong predictive capabilities (23).

#### Real-time surveillance and efficient monitoring

AI has also significantly improved real-time, continuous disease surveillance. Integrating AI with mobile data and syndromic surveillance systems has shown promise in enhancing outbreak detection and response, enabling immediate alerts for emerging public health threats (24, 25). Combining whole-genome sequencing (WGS) with machine learning for outbreak detection has revealed multiple outbreaks that traditional methods overlooked, showcasing AI’s potential to enhance surveillance systems (26, 27). Beyond detection, AI enhances predictive forecasting by integrating variables like geography, climate, and travel patterns. Machine learning models can analyze these datasets to identify regions at high risk for outbreaks, enabling proactive measures.

#### Efficiency in screening and reducing disease transmission

The AI-based system also reduces waiting time and minimizes bottlenecks and human interaction, significantly lowering the risk of disease transmission while ensuring efficient and accurate traveler screening (28). At Seychelles International Airport, the AI-based health biometric corridor enhances disease surveillance by processing each traveler in under one second. Screening about 30 passengers per minute and reducing processing times by 84%, the corridor allows a 300-passenger aircraft to be cleared in under 10 min (29).

Taken together, these studies indicate that AI effectiveness is highly conditional rather than uniform, and the reported performance figures must be read against the settings that produced them. Controlled, image-based classification tasks report the highest accuracies, for example, ensemble and deep-learning models applied to curated CT and X-ray datasets achieve accuracies approaching 98%, whereas models trained on routine field data in resource-limited settings perform considerably less well, as illustrated by the Senegalese COVID-19 prediction model, which achieved 73% accuracy and an AUROC of 0.69. Thermal-imaging approaches, despite their operational appeal for airport screening, have shown only limited sensitivity and specificity for detecting infection, underscoring that the modality most readily deployable at points of entry is also among the least validated for case detection. This gradient, from high performance in controlled conditions to markedly lower performance in real-world, low-resource environments, is the single most consistent pattern across the evidence, and it has direct implications for SSA airports, where data are often incomplete and screening must occur at speed. A further limitation is that the strongest performance evidence derives from retrospective or simulated datasets rather than prospective deployment, so reported accuracies likely represent an upper bound rather than expected field performance. The Seychelles biometric corridor is frequently cited as a proof of concept, yet it is documented in trade press rather than peer-reviewed evaluation, and its headline throughput metrics speak to processing efficiency rather than to diagnostic accuracy or outbreak-detection sensitivity. Critically interpreted, the literature therefore supports the claim that AI can enhance the speed and scale of surveillance, but provides much weaker support for claims about detection accuracy under the conditions that actually obtain at SSA airports.

### Ethical and privacy concerns

The deployment of AI in public health raises significant ethical and privacy concerns that must be addressed to ensure equitable, transparent, and trustworthy applications. These concerns are especially critical in sensitive areas such as disease surveillance, where the use of personal health data must balance the potential public health benefits with the protection of individual rights (30). For AI to be feasible and widely adopted, these ethical challenges must be mitigated through well-defined policies and frameworks that protect privacy, ensure fairness, and promote public trust in AI systems (31).

#### Privacy and data protection challenges

During the COVID-19 pandemic, AI-driven tracking tools effectively contained the virus, but often compromised privacy (32). Similarly, there was an emphasis on robust data protection measures to maintain patient agency. Regulatory measures were proposed, but the study acknowledged that regulation often lags behind technological advancements (33).

#### Equity and algorithmic bias

Ethical concerns in AI deployment extend beyond privacy to include equity, transparency, and algorithmic bias. A commentary on health equity stressed the importance of ensuring equitable access to AI technologies, particularly in underserved populations, to avoid exacerbating health disparities (34). Another study called for ethical oversight and transparency in AI applications, noting the lack of cohesive ethical frameworks across countries. Another study advocated for international collaboration to develop standardized guidelines addressing algorithmic bias, data privacy, and accountability (35).

#### Regulatory frameworks for AI in public health

A review of AI applications in COVID-19 management highlighted its role in enhancing disease detection and diagnosis. Still, it noted limited discussion on long-term implications, such as data privacy and ethical oversight. Comprehensive regulatory frameworks were recommended to address these concerns (36). While AI optimizes diagnostics and healthcare delivery, the lack of robust regulations poses risks to patient privacy and data security (37).

The WHO’s Ethics and Governance of Artificial Intelligence for Health framework emphasizes transparency, accountability, and bias mitigation in AI deployment, aligning with studies that highlight AI’s effectiveness in enhancing surveillance, particularly for real-time outbreak detection at international airports (38). However, this framework lacks specific guidelines for disease detection at critical entry points such as airports, where data privacy risks are elevated. To address this gap, ethical AI deployment must include enforceable guidelines for privacy protection and accountability, as well as regulatory clarity, as emphasized in the Regulatory Considerations on AI for Health report (2023) (39, 40).

A striking feature of the ethics literature is the degree of convergence it displays: across commentaries, reviews, and guidance documents, the same four concerns recur, privacy and data protection, algorithmic bias and equity, transparency and accountability, and the lag of regulation behind technology. This consensus is reassuring in that the relevant risks are well characterized, but it is also revealing of the literature’s principal weakness. The great majority of these contributions are conceptual or normative rather than empirical; as reflected in the included studies, many are explicitly limited by a lack of supporting data or of concrete case studies, and few test whether the safeguards they advocate are workable in practice. The result is broad agreement on principles coupled with thin evidence on implementation. This matters for the airport context in two specific ways. First, almost none of this work addresses points of entry, where the ethical calculus differs from clinical or community settings: consent is harder to obtain meaningfully from transiting passengers under time pressure, opt-out is constrained by the compulsory nature of border processing, and biometric data are captured in a security-sensitive environment. The WHO guidance on the ethics and governance of AI for health is the most authoritative reference point, yet, as we note above, it does not provide entry-point-specific guidance. Second, the equity concern is not merely abstract here: models developed and validated on non-African populations may systematically underperform for the travellers actually screened at SSA airports, so deploying them without local revalidation risks importing rather than mitigating bias. Critically synthesized, the ethics evidence thus offers a clear normative framework but leaves the operational and airport-specific questions substantially unanswered.

### Infrastructure and capacity

While AI has the potential to liberate the labor force by automating tasks and improving efficiency, its successful deployment in public health, especially in low- and middle-income countries (LMICs), still depends on robust digital infrastructure and adequate human capacity. The implementation of AI and digital technologies in these settings faces significant gaps in both infrastructure and skilled personnel, highlighting the need for capacity-building initiatives to ensure effective AI integration.

#### Infrastructure and skilled workforce gaps

A qualitative review of surveillance systems in LMICS highlighted key barriers, including resource limitations and a lack of trained personnel (41). Similarly, a scoping review of digital technologies for disease surveillance in Tanzania identified 13 technologies, many of which lacked interoperability and were hindered by fragmented implementation. The study emphasized the limited investment in community-based surveillance systems, further exacerbating the challenges of scaling up these technologies in resource-poor settings (42). An opinion piece on AI applications in Africa highlighted that while AI can improve diagnosis and healthcare accessibility, its implementation in resource-poor settings remains limited due to insufficient technical capacity and infrastructure (43, 44) These studies underscore the need for targeted investments in infrastructure and capacity building to deploy AI technologies in LMICs effectively.

#### Capacity building and training initiatives

The Policy Framework for AI in Tanzania’s Health Sector (2022) highlights capacity building as a key area for AI integration, but lacks specific strategies for implementing AI at border points like airports (45). Countries like Nigeria have addressed workforce limitations through short, intensive training programs for healthcare workers. Nigeria’s SORMAS project, which focused on AI-assisted disease surveillance, increased AI tool utilization from 18 to 91% after a one-week digital surveillance boot camp. This model, which involves task-shifting to auxiliary health workers, is a viable strategy to address skill gaps and enhance the efficiency of AI deployments in airport settings (46).

Read across these studies, a consistent message emerges that reframes the feasibility question itself: the binding constraint on AI surveillance in LMIC and SSA settings is rarely the sophistication of the algorithm and almost always the surrounding ecosystem of data, connectivity, interoperability, and human capacity. The scoping evidence from Tanzania is instructive identifying numerous deployed technologies that nonetheless failed to deliver because they lacked interoperability and were implemented in a fragmented manner, and it stands in productive contrast to the Nigerian SORMAS experience, where a brief, intensive training intervention raised tool utilization from 18 to 91%. Juxtaposed, these two cases suggest that the determinants of success are organizational and human as much as technical: tools fail when they are bolted on without integration, and succeed when accompanied by workforce investment and task-shifting. This interpretation is important because it shifts the locus of feasibility away from procuring advanced AI and toward building the conditions in which any AI can function. For airports specifically, it implies that the most consequential early investments are not in novel models but in reliable connectivity and power, interoperable data standards that allow an AI layer to communicate with existing entry-screening and health-information systems, and short-cycle training for the staff who will operate the systems across shifts. The evidence also exposes a gap in the literature itself: infrastructure and capacity studies are typically situated in clinical or community settings, and their lessons are here extrapolated to the airport environment rather than tested in it, an extrapolation that is reasonable but unproven, and that further motivates the pilot studies recommended below.

## Discussion

The implementation of AI-driven disease surveillance systems at SSA international airports holds significant promise but is challenged by issues related to infrastructure, data quality, and capacity (47). AI has demonstrated the ability to improve disease detection accuracy and response times, particularly in resource-constrained environments. Despite these successes, the broader implementation of AI in SSA faces key obstacles, including the need to enhance data collection systems to ensure high-quality, real-time data and the requirement for AI-specific infrastructure to support these advanced technologies (48). Addressing ethical concerns, such as privacy, data security, and algorithmic bias, is essential to gain public trust and ensure equitable access to AI tools. Furthermore, overcoming capacity-building challenges, particularly in training the local workforce, is vital, as many SSA airports currently lack the skilled personnel needed to operate and manage AI-driven systems effectively. Political and financial support from both local governments and international organizations is critical to securing the necessary investments for AI deployment (49). In addition, comprehensive policy frameworks are necessary to regulate the use of AI in disease surveillance, ensuring compliance with privacy laws and facilitating cross-sector collaboration for long-term sustainability.

The recommended implementation framework outlined in Table 2 below focuses on addressing critical infrastructure, capacity, ethical, privacy, and policy challenges to ensure successful deployment and long-term sustainability. The Infrastructure and Capacity section emphasizes leveraging existing resources, such as integrating AI with current airport health technologies (e.g., thermal scanning, mobile data) and using cloud-based AI solutions to minimize initial hardware investments and maximize scalability across airports. This approach aligns with findings from studies in resource-limited settings, where cloud computing has been identified as a cost-effective means of supporting AI systems (50). Additionally, prioritizing open-source AI tools reduces the financial burden of licensing fees while fostering local innovation, which is crucial given the resource constraints in many SSA countries. The Ethical and Privacy section proposes clear guidelines for data privacy, ensuring compliance with global standards such as the General Data Protection Regulation (GDPR) and implementing anonymization protocols to protect passenger health data. These privacy protections are vital, as previous studies on AI in public health have highlighted privacy concerns during the COVID-19 pandemic, where data misuse and lack of transparency were major obstacles to the public’s trust (51). Furthermore, establishing AI accountability through regular algorithmic audits and transparency reports ensures that AI systems function fairly and effectively, addressing concerns of algorithmic bias that have emerged in other health-related AI applications. This approach provides a clear, low-cost strategy for AI integration, offering a pathway to enhance public health surveillance systems while addressing the unique challenges faced by SSA airports.

**Table 2:** Recommended implementation framework.

Category	Recommendation	Implementation details	Timeframe	Evidence basis
Infrastructure and Capacity	Leverage existing infrastructure and cloud-based solutions	Integrate AI with existing airport health systems (e.g., thermal scanners, mobile data) and use cloud platforms for processing and storage, avoiding costly local hardware and data Centres while allowing the system to scale with demand.	Short-term	Evidence-based (50)
	Prioritize open-source AI tools	Use open-source AI models and software with clear documentation to reduce licensing costs, foster local adaptation, and improve transparency of predictions.	Short-term	Author-proposed
	Build workforce capacity through short-cycle training	Implement affordable, short-term training for airport staff using online platforms and task-shifting to auxiliary workers, drawing on models such as Nigeria’s SORMAS boot camp.	Short- to medium-term	Evidence-based (42, 46)
	Collaborate with universities and NGOs	Partner with local universities to develop talent and with NGOs for resources and co-funding, building durable local innovation capacity.	Medium-term	Author-proposed
Ethical and Privacy	Use anonymized data and protect privacy by design	Anonymize personal data before analysis and embed basic data-protection and consent-management practices aligned with global standards (e.g., GDPR), reducing privacy risk while preserving surveillance value.	Short-term	Evidence-based (32, 33, 51)
	Ensure algorithmic transparency and auditability	Favour explainable, well-documented algorithms and schedule regular bias and performance audits to reduce hidden bias in detection and decisions.	Medium-term	Evidence-based (35, 38)
Deployment approach	Adopt incremental, pilot-based rollout	Begin with small-scale, low-cost pilots at one or two airports using non-invasive applications (e.g., temperature screening), validate throughput, reliability, privacy controls, and governance, then add more complex features (e.g., predictive models) as systems mature.	Short- to long-term	Author-proposed

### Operational realities at SSA airports

Translating these findings into practice requires attention to the operational environment of SSA airports, which differs markedly from the controlled research settings in which most of the reviewed AI systems were developed. Passenger throughput is a primary constraint: major SSA hubs must process arriving aircraft within tight turnaround windows, and any AI-assisted screening must operate at sub-minute speeds per passenger during peak flows to avoid creating bottlenecks at immigration and customs. The Seychelles biometric corridor, which processes travelers in under 1 s, illustrates the throughput threshold that any SSA deployment must meet to be operationally viable. Second, the physical infrastructure on which AI depends is frequently unreliable: intermittent mains power and variable broadband connectivity at many SSA airports threaten the continuous, real-time operation that AI surveillance assumes, making local edge computing, offline-capable models, and backup power essential design considerations rather than optional extras. Third, point-of-entry surveillance sits within a fragmented governance landscape in which public health ministries, civil aviation authorities (for example, the Tanzania Civil Aviation Authority and Tanzania Airports Authority), immigration services, and communications regulators each hold partial mandates; successful deployment therefore depends on inter-agency data-sharing agreements and clear lines of accountability rather than on technology alone. Fourth, rather than replacing existing International Health Regulations (2005) entry-screening workflows, an AI layer would most realistically augment them, adding automated anomaly detection and predictive flagging on top of established thermal screening and traveler health-declaration processes. Finally, sustained operation requires trained personnel available across all operating shifts, alongside data-handling protocols that satisfy aviation-security regimes governing the movement and storage of passenger information. These operational realities reinforce the case for staged, pilot-based implementation, in which throughput, reliability, governance, and workforce requirements are validated at one or two airports before any wider rollout.

### Lessons from existing point-of-entry implementations

The most directly relevant implementation to date is the AI-enabled health biometric corridor introduced at Seychelles International Airport in 2021, reported to be the first of its kind in Africa and the second worldwide. Designed by a border-security technology provider and fully integrated with the national travel-authorization system, the corridor combines temperature checks, facial-recognition matching against an approved-traveler database, and automated risk classification based on pre-departure health declarations, processing arriving travelers on the move without requiring them to stop. Its principal reported strengths are operational rather than epidemiological: high throughput, contactless processing, and seamless integration with an existing digital health-authorization platform. Importantly, however, peer-reviewed evaluation of the corridor’s public-health outcomes, such as the number of infections detected, false-positive and false-negative rates, or measurable impact on importation of disease, has not, to our knowledge, been published; the available accounts are operational descriptions and vendor and government communications. This absence of independent outcome evaluation is itself an important finding, and it tempers the confidence with which the Seychelles experience can be cited as evidence of feasibility. A central lesson for SSA is therefore that the integration of AI screening with a pre-existing traveler health-authorization system was the practical enabler of the Seychelles deployment, and that any comparable initiative should build in independent outcome measurement from the outset rather than relying on operational metrics alone.

Evidence from comparable point-of-entry screening programs provides a more sobering, but equally instructive, perspective on feasibility. A study of airport health screening at Fuzhou Changle International Airport in China, analyzing 559 travel-related infections identified between 2016 and 2019 (of which 94.3% were imported), reported an overall screening effectiveness of only 23.7% in detecting travel-associated infections (52). Consistent with this, rapid and systematic reviews of entry screening during the COVID-19 pandemic concluded that temperature- and thermal-based entry screening was unlikely, on its own, to be effective in preventing importation, because many infections are asymptomatic or in the incubation period at the time of travel. Two lessons follow for SSA. First, conventional screening modalities, particularly the thermal scanning on which many SSA airports currently rely, have limited sensitivity, which is precisely the gap that multimodal AI (for example, combining travel history, syndromic data, and risk stratification) is intended to address; the value proposition for AI rests on improving over this low baseline rather than merely automating it. Second, the modest measured effectiveness of even well-resourced screening programs underscores that AI-driven surveillance should be positioned as one layer within a broader detection system, with realistic expectations of incremental gain, rather than as a standalone solution. Taken together, these implementations suggest that AI surveillance at SSA airports is plausible and potentially valuable, but that its benefits must be demonstrated through carefully evaluated pilots rather than assumed from the operational success of early deployments.

The recommended AI-Specific Policy Framework for Sub-Saharan Africa (SSA) international airports, as outlined in Table 3, focuses on the responsible, secure, and equitable deployment of AI-driven disease surveillance systems. Key policies emphasize data privacy and protection, ensuring compliance with global standards such as GDPR, anonymizing passenger data, and establishing clear guidelines for data retention and disposal, thereby addressing privacy concerns raised during previous health crises. Transparency and accountability are crucial, requiring regular audits of AI systems to ensure fairness and mitigate bias, as seen in public health applications (53). Equity policies ensure that AI systems are accessible to all passengers and offer alternative methods for those who opt out. Security policies prioritize compliance with aviation standards and robust cybersecurity measures. Regulatory oversight calls for an independent body to monitor AI implementations, ensuring compliance with local and international regulations and encouraging cross-border collaboration to maintain consistent standards. Funding is secured through national health and transportation budgets, with public-private partnerships for financial sustainability. Finally, public engagement through transparency initiatives, educational campaigns, and grievance mechanisms builds trust and ensures the successful integration of AI technologies into SSA airports.

**Table 3:** Recommended AI-specific policy framework for SSA international airports.

Policy area	Recommendation	Implementation details	Timeframe	Evidence basis
Data Privacy and Protection	Establish data-privacy, consent, and retention policies	Align with global standards (e.g., GDPR) to safeguard passenger health data; obtain informed consent before collection (e.g., biometric scans, temperature checks) with a means to withdraw it; and set clear limits on data storage, with secure disposal after a defined retention period.	Short-term	Evidence-based (32, 33, 51)
AI Accountability and Transparency	Mandate explainability, independent audits, and clear accountability	Require surveillance algorithms to be explainable, with mechanisms for passengers to understand how decisions are reached; commission regular independent audits of accuracy, fairness, and bias; and assign clear responsibility for AI outcomes across providers and airport authorities, particularly where harm or error occurs.	Medium-term	Evidence-based (35, 38)
Equity and Accessibility	Ensure equitable, non-discriminatory access with opt-out alternatives	Subject AI systems to regular bias and equity testing to ensure they perform across diverse populations, and provide non-AI screening alternatives for passengers who opt out or lack access to the required technology.	Medium-term	Evidence-based (34, 35)
Security and Safety	Comply with aviation security and AI cybersecurity standards	Align systems with aviation security regulations (e.g., ICAO standards) for secure data transmission and system integrity, and adopt an AI-specific cybersecurity policy covering encryption, access control, and threat detection.	Short- to medium-term	Author-proposed
Regulatory Oversight and Governance	Provide phased oversight and cross-border coordination	Establish or designate an independent body to oversee AI surveillance at airports; adopt a phased regulatory approach that permits low-risk pilots before scaling; and coordinate with international bodies (e.g., WHO, ICAO) toward standards that can be adopted across SSA airports.	Medium- to long-term	Author-proposed (40)
Funding and Sustainability	Secure sustainable, mixed-source funding	Incorporate AI surveillance as a recurrent line item in national health and transport budgets, and create incentives (e.g., tax relief, grants, subsidies) for private-sector provision and co-funding.	Long-term	Author-proposed
Public Engagement and Trust	Build public awareness and accessible redress	Use low-cost channels (e.g., social media, airport signage) to explain the benefits of AI surveillance and its privacy safeguards, and provide a simple, accessible mechanism for passengers and staff to report privacy violations, data misuse, or system malfunctions.	Short-term	Author-proposed

### Strength of the study

This study’s strength lies in its focus on integrating artificial intelligence into real-time disease surveillance at airports, an underexamined aspect of disease surveillance. By concentrating on high-traffic entry points such as international airports in SSA, it offers a potentially practical approach to early outbreak detection and provides preliminary, actionable recommendations for policymakers and practitioners.

## Limitations of the study

A key limitation of the evidence is the generalizability of findings, as successful AI implementations in countries like Senegal and Seychelles may not apply to other SSA countries due to differences in healthcare infrastructure, data quality, and regional contexts. Additionally, few studies specifically explore AI for disease surveillance in SSA. Despite promising results elsewhere, limited SSA research hampers understanding of AI’s role in disease monitoring and early warning systems. Future research should focus on pilot projects in SSA to explore AI’s potential in real-world disease surveillance at airports, addressing region-specific barriers. Relatedly, almost none of the included studies evaluated AI surveillance at airports specifically, and none did so at SSA airports; feasibility is therefore inferred from analogous healthcare and public-health surveillance settings rather than demonstrated directly in the point-of-entry context. The conclusions of this review should accordingly be interpreted as evidence-informed hypotheses requiring site-specific validation, rather than as confirmation of operational feasibility at any particular airport.

Exclusion of studies published in languages other than English. As a result, relevant studies in non-English languages may have been overlooked, potentially introducing language bias. Future reviews could consider including studies in other languages, provided translation resources are available, to ensure a more comprehensive analysis of AI applications in disease surveillance.

## Conclusion

This study suggests that AI-driven disease surveillance systems have considerable potential to enhance public health security at Sub-Saharan African (SSA) international airports, and proposes a preliminary framework for addressing infrastructure, data privacy, and regulatory challenges. Because the supporting evidence is largely conceptual and policy-oriented rather than derived from empirical deployment at airports, these conclusions should be regarded as evidence-informed hypotheses rather than established findings. Future research should therefore priorities assessing country-specific feasibility through pilot studies that evaluate real-world implementation, identify best practices, and address challenges such as resource constraints, regulatory barriers, and infrastructure gaps. Such studies would be central to refining AI models and ensuring they are adaptable to the differing needs of individual SSA countries. The work also has broader implications for global health, since AI systems at critical entry points may improve early detection, emergency response, and overall health system resilience, both within SSA and beyond. If validated, these findings could inform health system strengthening and offer transferable lessons for other low- and middle-income regions, contributing to the development of more equitable, data-driven health infrastructures.
